# Loss of skin elasticity is associated with pulmonary emphysema, biomarkers of inflammation, and matrix metalloproteinase activity in smokers

**DOI:** 10.1186/s12931-019-1098-7

**Published:** 2019-06-24

**Authors:** Michael E. O’Brien, Divay Chandra, Robert C. Wilson, Chad M. Karoleski, Carl R. Fuhrman, Joseph K. Leader, Jiantao Pu, Yingze Zhang, Alison Morris, Seyed Nouraie, Jessica Bon, Zsolt Urban, Frank C. Sciurba

**Affiliations:** 1 Division of Pulmonary Allergy and Critical Care Medicine Department of Medicine, University of Pittsburgh School of Medicine and University of Pittsburgh Medical Center, Kaufmann Building, Suite 1211, 3471 Fifth Ave, Pittsburgh, PA 15213 USA; 2 Department of Radiology, University of Pittsburgh, Pittsburgh, PA USA; 3 Department of Immunology, University of Pittsburgh, Pittsburgh, PA USA; 4 Veterans Affairs Pittsburgh Healthcare System, Pittsburgh, PA USA; 5 Department of Human Genetics Graduate School of Public Health, University of Pittsburgh, Pittsburgh, PA USA

**Keywords:** COPD, Emphysema, Skin, Elasticity, Metalloprotease, Inflammation

## Abstract

**Background:**

Elastin breakdown and the resultant loss of lung elastic recoil is a hallmark of pulmonary emphysema in susceptible individuals as a consequence of tobacco smoke exposure. Systemic alterations to the synthesis and degradation of elastin may be important to our understanding of disease phenotypes in chronic obstructive pulmonary disease. We investigated the association of skin elasticity with pulmonary emphysema, obstructive lung disease, and blood biomarkers of inflammation and tissue protease activity in tobacco-exposed individuals.

**Methods:**

Two hundred and thirty-six Caucasian individuals were recruited into a sub-study of the University of Pittsburgh Specialized Center for Clinically Orientated Research in chronic obstructive pulmonary disease, a prospective cohort study of current and former smokers. The skin viscoelastic modulus (VE), a determinant of skin elasticity, was recorded from the volar forearm and facial wrinkling severity was determined using the Daniell scoring system.

**Results:**

In a multiple regression analysis, reduced VE was significantly associated with cross-sectional measurement of airflow obstruction (FEV1/FVC) and emphysema quantified from computed tomography (CT) images, β = 0.26, *p* = 0.001 and β = 0.24, p = 0.001 respectively. In emphysema-susceptible individuals, elasticity-determined skin age was increased (median 4.6 years) compared to the chronological age of subjects without emphysema. Plasma biomarkers of inflammation (TNFR1, TNFR2, CRP, PTX3, and SAA) and matrix metalloproteinase activity (MMP1, TIMP1, TIMP2, and TIMP4) were inversely associated with skin elasticity.

**Conclusions:**

We report that an objective non-invasive determinant of skin elasticity is independently associated with measures of lung function, pulmonary emphysema, and biomarkers of inflammation and tissue proteolysis in tobacco-exposed individuals. Loss of skin elasticity is a novel observation that may link the common pathological processes that drive tissue elastolysis in the extracellular matrix of the skin and lung in emphysema-susceptible individuals.

**Electronic supplementary material:**

The online version of this article (10.1186/s12931-019-1098-7) contains supplementary material, which is available to authorized users.

## Background

Cigarette smoking is the most important risk factor for the development of chronic obstructive pulmonary disease (COPD) in the developed world and is a major cause of premature morbidity and mortality [1]. Comorbidities associated with COPD, such as osteoporosis, cardiovascular disease, and skeletal muscle atrophy, appear to correlate with the parenchymal emphysema dominant pattern of lung disease, and with a prevalence disproportionate to what would be expected in a similar tobacco-exposed population [2–5]. Systems biology approaches, which incorporate computational modelling of specific genetic, molecular, and cellular processes, have provided new perspectives on the mechanisms that drive the varying manifestations of pulmonary and multisystem disease in COPD [6, 7]. Identification of important pathogenic mechanisms that drive pulmonary and comorbid illness in COPD, including systemic inflammation, oxidative stress, and protease imbalance, have helped inform novel therapeutic advances in COPD [8, 9]. However an individual’s susceptibility to the complex pathogenic mechanisms underlying the development of airflow obstruction, emphysema, and associated systemic comorbid findings in smokers remains incompletely understood [10, 11].

Cutaneous manifestations of COPD affecting skin texture, thickness, and connective tissue integrity have been historically described [12]. More recent reports indicate that an independent association exists between facial wrinkling and airflow obstruction, which suggests that the lung and skin share common susceptibility to the deleterious effects of tobacco smoke exposure [13]. Facial wrinkle formation results from immobilization of transient wrinkles due to loss of skin elasticity and is influenced by aging, ultraviolet light exposure, and smoking [14, 15]. Furthermore, tobacco smoke exposure is known to be an independent risk factor for premature facial wrinkling [16–18]. In smokers with COPD, elastin degradation in the skin is associated with emphysema severity and carotid pulse wave velocity, indicating that systemic elastin breakdown may be increased in susceptible individuals and not confined to the lung alone [19]. Skin elasticity, a key biomechanical property of skin, decreases significantly with age, is quantifiable, and changes in disease states caused by genetic disruption of elastic fibers, such as cutis laxa [20]. Taken together, alterations in skin elasticity may represent a novel non-invasive measure of the systemic effects that alter the extracellular matrix (ECM) in response to tobacco smoke in COPD.

We evaluated skin elasticity and facial wrinkling in a prospective cohort of well characterized current and former smokers. We hypothesized that skin elasticity would correlate with the severity of pulmonary disease in susceptible tobacco-exposed individuals and be associated with systemic markers of inflammation and tissue protease imbalance.

## Methods

### Study population

Study participants (*n* = 236) were recruited prospectively into a sub-study from the University of Pittsburgh Specialized Center of Clinically Orientated Research (SCCOR) cohort, which includes current and former smokers aged > 40, with a minimum 10 pack-year tobacco exposure residing around southwestern Pennsylvania [21]. Participants completed demographic and medical history questionnaires including self-reported average weekly sunlight exposure. All data acquisition procedures were performed under a University of Pittsburgh Institutional Review Board-approved protocol with written informed consent obtained from all participants.

### Skin elasticity measurement

Skin elasticity measurements were performed on the volar forearm of each subject using the Dermalab® (Cortex Technology, Denmark) skin elasticity unit as previously described [20, 22] (see Additional file 1: Figure S1). The volar forearm was chosen to reduce the confounding influence of photoaging as a consequence of ultraviolet light exposure. In brief, incremental negative pressure was applied using a 10 mm diameter suction cup secured midway along the volar forearm using a double-sided adhesive tape until the section of skin was raised 1.5 mm (Δx). Measured variables include the pressure difference required to lift the skin (ΔP), and the time the skin takes to return to its original position upon release of the vacuum (retraction time, RT). The mean of ten values per subject, taken from five locations on each forearm, was used for analysis. Young’s elastic modulus (E) was calculated by the DermaLab® software by solving the following equation: Δx = Ψ * ΔP * r^4^ / (E * s^3^), where Δx and ΔP are as described above, Ψ is an instrument constant, r is the radius of the skin patch displaced (0.005 m), and s is the estimated thickness of the skin (1 mm). The viscoelastic modulus (VE) was computed by the following formula: VE = E / RT_n_, where RT_n_ is a normalized RT value obtained by dividing the measured RT value with average control RT of 260 ms.

### Facial wrinkling score

**Fig. 1:**
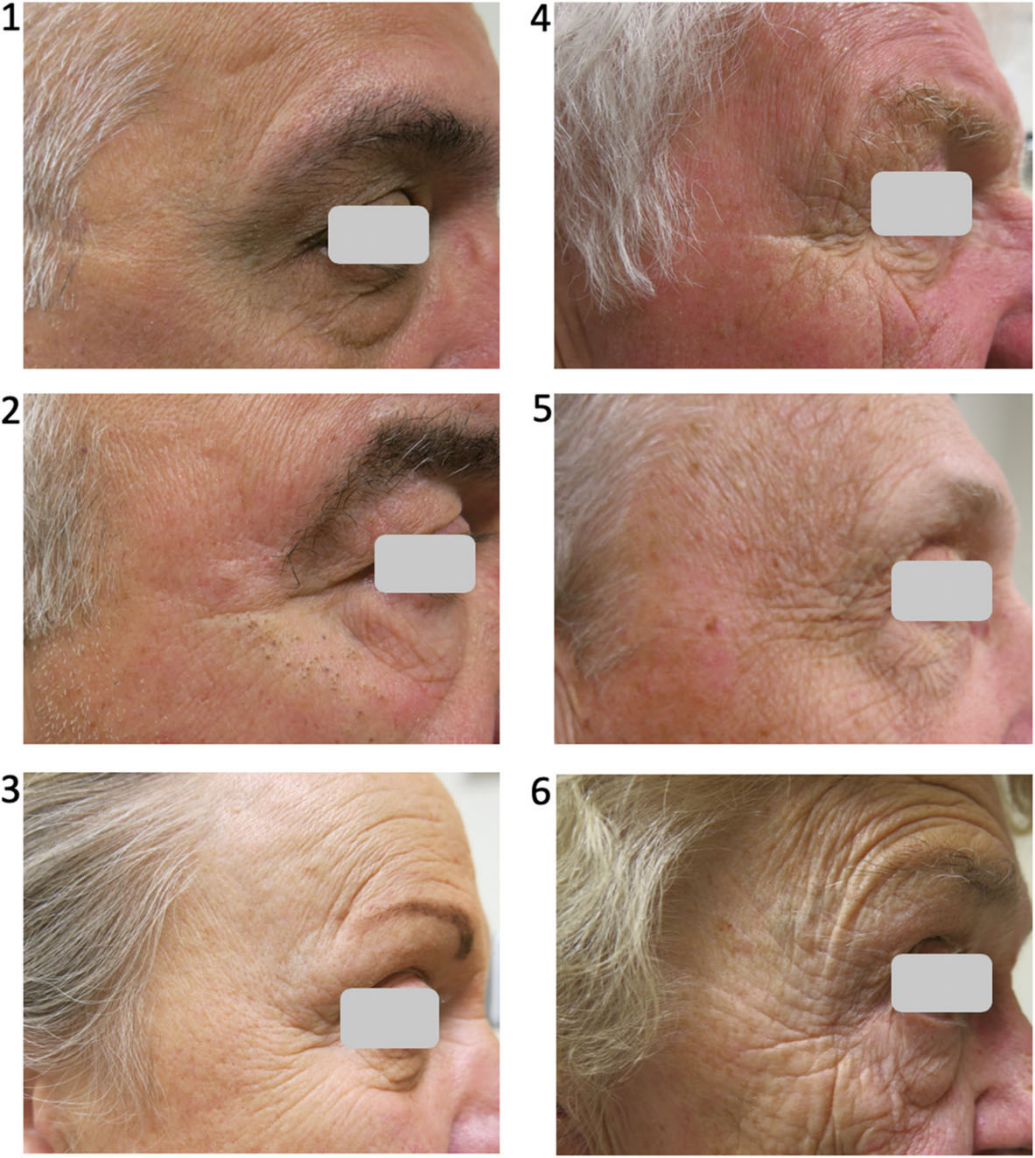
Measurement of facial wrinkling. Daniell system for scoring the appearance of facial wrinkles in the crow’s-foot area: 1) Essentially unwrinkled. 2) Between 2 and 6 wrinkles ≤3 cm in length. 3) Several prominent wrinkles 3-4 cm in length. 4) Wrinkles ≥5 cm in length, may extend onto cheek area. 5) Prominent wrinkles extending from crow’s-foot area over cheeks and forehead. 6) Profound wrinkling over most of the face

Photographs were taken of all subjects using the same camera, subject distance from the camera, and lighting conditions. The left and right temporal regions, forehead and peri-oral region were photographed to assess facial wrinkling. Facial wrinkling was determined from the left and right temporal (Crow’s foot) region using the Daniell scoring system, a validated scale from 1 to 6 (1-unwrinkled; 2- < 6 wrinkles ≤3 cm in length; 3-wrinkles > 3 cm but < 5 cm; 4-wrinkles ≥5 cm; 5-wrinkles > 5 cm, over cheeks and forehead; 6-profound wrinkling) [23] (Fig. 1). Two independent observers, blinded to the subjects’ clinical information, rated left and right temporal wrinkling and reported a mean value. The average facial wrinkling score (FWS) of the two observers was used for analysis. Severe wrinkling was defined as a Daniell score ≥ 4 as previously described [13].

### Clinical phenotype

Post-bronchodilator spirometry, lung volumes by body plethysmography, and single breath diffusing capacity of the lung for carbon monoxide (DLCO) were performed in each study participant according to American Thoracic Society standards [24] using standard reference eqs. [24–26]. Emphysema was quantified from chest computed tomography (CT) images acquired on multi-detector scanners with subjects holding their breath at end-inspiration. After segmenting the lung from the CT images, the Hounsfield unit (HU) value designating the lower 15th percentile of the HU value histogram (Perc15) and the percentage of lung voxels below − 950 HU (%LAA) were computed to quantify emphysema. A single radiologist, blinded to subject identities and other characteristics, visually assessed emphysema severity using a previously validated, 6-point semi-quantitative scoring system (0 = none, 1 = trace/minimal, 2 = mild, 3 = moderate, 4 = severe, 5 = very severe, corresponding to 0, < 10%, 10–25%, 26–50%, 51–75, and > 75% visual emphysema) [27]. For non-continuous analysis, COPD was defined using FEV1/FVC less than 70%. and subjects were categorized as having no emphysema based on a visual emphysema score of zero.

### Biomarker measurement

Plasma and serum were drawn from participants using standardized phlebotomy procedures, then separated immediately by centrifugation and frozen for later analysis. A bead-based immunoassay was performed for quantification of tumor necrosis factor α (TNFα), TNF receptor 1 (TNF-R1), and TNF-R2 (Invitrogen). Matrix metalloproteases (MMP) and tissue inhibitors of metalloproteases (TIMP), including MMP1, MMP7, TIMP1, TIMP2, and TIMP4, were assayed using Performance Assay Luminex kits (R&D Systems). C reactive protein (CRP) and serum amyloid A (SAA) were measured using V-Plex assay kit (MSD). Pentraxin 3 (PTX3) was measured using DuoSet enzyme-linked immunoassays (R&D Systems).

### Statistical analysis

Bland-Altman analysis was performed to determine agreement between the two independent observers scoring facial wrinkling severity. Bivariate and multiple regression models were used to determine relationships between facial wrinkling score (FWS) and skin viscoelastic modulus (VE) with spirometric measures of airflow obstruction, CT-assessed emphysema, and biomarkers of matrix-metalloprotease activity. Adjustment for covariates included age, gender, self-reported sunlight exposure, current smoking status, and self-reported pack-year smoking history. Analyses were carried out with Stata v13.0 (StataCorp, Texas), and graphs were generated using Prism v6 (GraphPad Software, Inc., California). Relationships were reported using the standardized coefficient (β) and statistical significance was determined as a two-tailed *P*-value < 0.05.

## Results

### Subject characteristics

All subjects (*n* = 236) were Caucasian with a mean age of 70.4 ± 5.9 years, they were all current or prior smokers and gender was equally balanced with 123 (52.1%) males (Table 1). One hundred and thirteen (47.9%) subjects had COPD based on spirometry (FEV1/FVC < 0.70) with severity being nearly equally distributed between GOLD Stage I to IV COPD. Emphysema was detected by visual inspection of CT images in 130 (55.1%) subjects.

**Table 1:** Subject Characteristics

Total subjects (*N*)	236
Age, mean ± SD	70.4 ± 5.9
Non-Hispanic white, n (%)	236 (100)
Male gender, n (%)	123 (52.1)
BMI, mean ± SD	28.4 ± 4.5
Pack years, median (IQR)	48 (37.5–70)
Current smoker, n (%)	74 (31.4)
FWS, median (IQR)	3 (2.0 to 3.5)
Wrinkled, n (%)	46 (19.5)
VE, median (IQR)	2.5 (1.8 to 3.4)
FEV1% predicted, mean ± SD	82.6 ± 22.4
FEV1/FVC%, mean ± SD	67.7 ± 13.3
FEV1/FVC < 70%, n (%)	113 (47.9%)
GOLD stage, n (% of total N)	
0 (Non-obstructed)	123 (52.1%)
I	31 (13.2%)
II	23 (9.8%)
III	36 (15.3%)
IV	20 (8.5%)
DLCO % predicted, mean ± SD	67.2 ± 18.0
RV % predicted, mean ± SD	120.5 ± 33.8
RV/TLC %, mean ± SD	44.2 ± 9.0
Visual emphysema, n (%)	130 (55.1%)
%LAA, mean ± SD	4.0 ± 8.2
Perc15, mean ± SD	− 910.9 ± 26.9

Notes: BMI, body mass index; SD, standard deviation; FWS, facial wrinkling score; IQR, interquartile range; VE; skin viscoelasticity modulus; FEV1, forced expiratory volume in 1 s; FVC, forced vital capacity in 1 s; GOLD, Global initiative for obstructive lung disease; DLCO, diffusion capacity for carbon monoxide; RV, residual volume; TLC, total lung capacity; LAA% = low attenuation areas, lung voxels with Hounsfield Unit (HU) values less than − 950; Perc15, HU value at the 15th percentile of the HU value histogram of lung voxels

### Skin elasticity correlates with Daniell facial wrinkling score

Bland-Altman analysis of facial wrinkling scores found excellent agreement between the two observers with a mean difference of − 0.063 (95% limits of agreement: − 1.25 to 1.12), intraclass correlation coefficient (ICC) of average for one-way random effects: 0.94 (95% confidence interval 0.93–0.96). There was a significant inverse correlation between VE and the facial wrinkling score, indicative of their biological association (β = − 0.29, *P* < 0.001).

### Bivariate analysis of skin elasticity and baseline demographics

As anticipated, VE was inversely correlated with age (β = − 0.49, *P* < 0.0001). Male gender (β = − 0.16, *P* = 0.017) and longer pack-year smoking history (β = − 0.20, *P* = 0.002) were also associated with lower VE, while increased BMI was associated with a higher VE (β = 0.46, *P* < 0.0001) (Table 2). The association between self-reported weekly sun exposure and VE was not significant (*P* = 0.18). Bivariate analysis of facial wrinkling revealed a significant association with age (β = 0.25, *P* < 0.001) and BMI (β = − 0.15, *P* = 0.025), but not with self-reported sun exposure (*P* = 0.72).

**Table 2:** The skin viscoelastic modulus correlates with facial wrinkling, age, sex, body mass index, and pack years in current or former smokers

	Facial Wrinkling Score (FWS)		Viscoelastic Modulus (VE)	
	β	*P*-value	β	*P*-value
VE	− 0.29	<*0.0001*	–	*–*
Age	0.25	*0.0001*	−0.49	*<0.0001*
Male sex	0.05	0.41	−0.16	*0.0167*
BMI	−0.15	*0.025*	0.46	*<0.0001*
Current smoker	−0,01	0.85	0.03	0.63
Pack Year Smoking	−0.05	0.48	− 0.20	*0.0018*
Sun Exposure	−0.02	0.74	−0.09	0.19

Notes. Bivariate regression analysis was performed to test the correlation between facial wrinkling and skin elasticity and exposure variables in the study cohort. BMI, body mass index

### Skin elasticity correlates with cross-sectional measures of lung function

Bivariate and multiple regression analysis of VE following adjustment for covariates (age, gender, sunlight exposure, current smoking and pack-year smoking history) demonstrated a significant relationship with measures of spirometry, gas transfer, air trapping, and quantitative emphysema (Perc15, %LAA) (Table 3). FEV1/FVC was strongly associated with lower VE measurements, in contrast FEV1 did not correlate with skin elasticity in the bivariate analysis; however, a significant association did emerge after correction for covariates. There was no association in the bivariate or multiple regression analyses between FWS and cross-sectional FEV1, FEV1/FVC, RV/TLC, or quantitative emphysema severity. Notably, facial wrinkling was significantly associated with DLCO (β = − 0.13, *P* = 0.04), the physiologic correlate most linked with emphysema severity, and remained significant in the adjusted analysis (Table 3).

**Table 3:** The skin viscoelastic modulus is an independent predictor of pulmonary emphysema and airflow obstruction

	Bivariate		Multiple Regression^a^	
	β	*P*-value	β	*P*-value
%LAA				
VE	*−0.23*	*0.0004*	−0.26	*0.003*
FWS	0.11	0.09	0.14	0.11
Perc15				
VE	0.17	*0.009*	0.24	*0.001*
FWS	− 0.04	0.58	−0.06	0.39
FEV1 (%pred)				
VE	0.09	0.164	0.18	*0.013*
FWS	−0.02	0.73	−0.07	0.35
FEV1/FVC (%pred)				
VE	0.24	*0.0003*	0.26	*0.001*
FWS	−0.11	0.08	−0.13	0.07
DLCO (%pred)				
VE	0.38	*<0.0001*	0.35	*<0.0001*
FWS	*−0.13*	*0.04*	−0.13	*0.038*
RV/TLC				
VE	−0.28	*<0.0001*	−0.16	*0.03*
FWS	0.01	0.09	0.06	0.35

Notes. DLCO, diffusion capacity for carbon monoxide; FEV1, forced expiratory volume in 1 s; FWS, facial wrinkling score; FVC, forced vital capacity in 1 s; LAA% = low attenuation areas, tissue voxels with Hounsfield Unit attenuation values less than −950; Perc15, cut off value in Hounsfield units below which 15% of all voxels are distributed; RV, residual volume; TLC, total lung capacity; VE; skin viscoelastic modulus

^a^Adjusted for age, gender, sun exposure, active smoking and pack year smoking history

### Loss of skin elasticity is associated with an increased likelihood of pulmonary emphysema

**Fig. 2:**
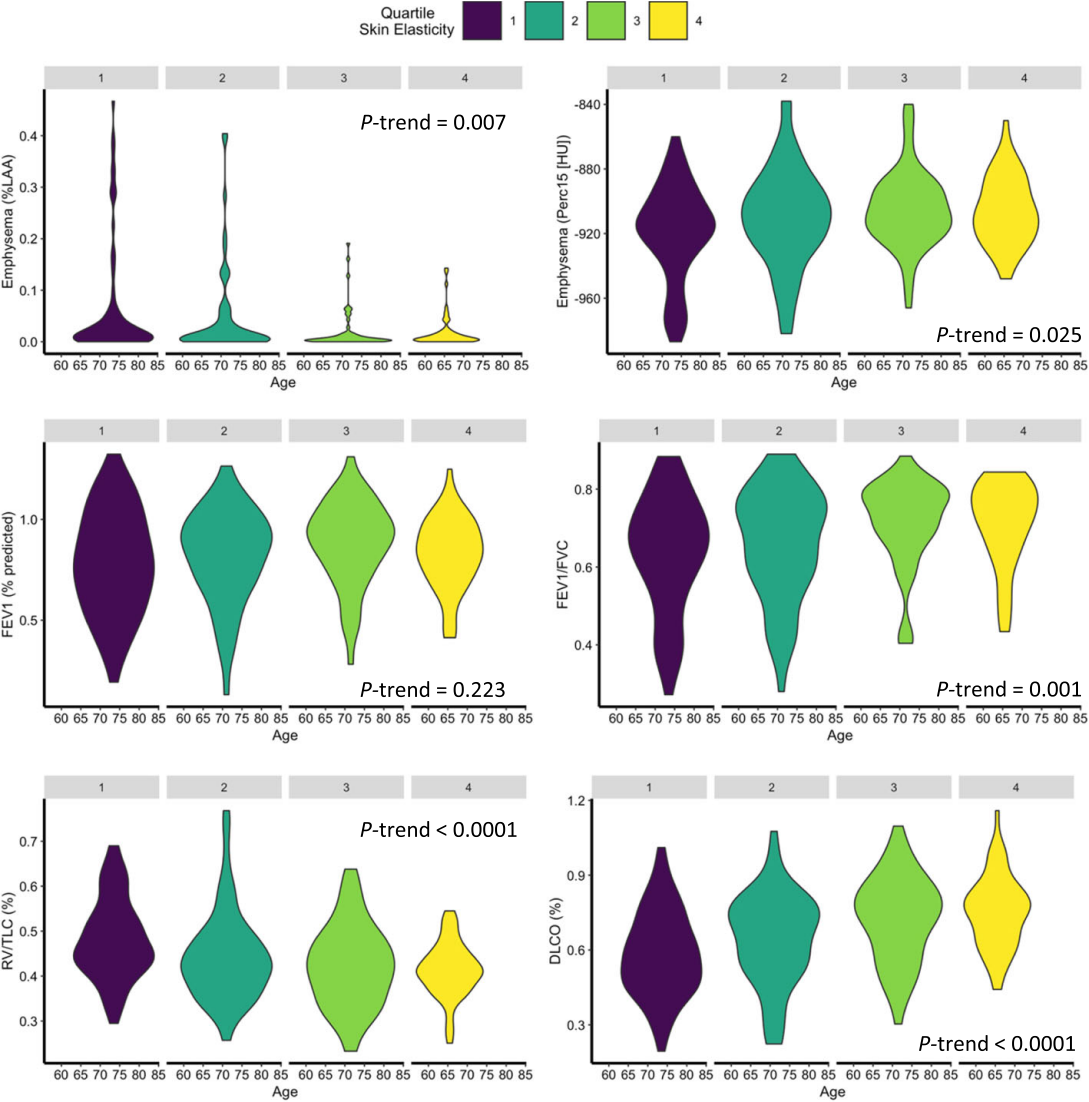
Loss of skin elasticity is associated with greater impairment in lung function and increased pulmonary emphysema. Skin elasticity was associated with pulmonary emphysema determined by %LAA and Hist15. Skin elasticity was not associated with impairment in FEV1, however a significant association was observed between quartiles of skin elasticity and the severity of airflow obstruction, diffusion impairment, lung hyperinflation (RV/TLC) and pulmonary emphysema. Legend: FEV1, forced expiratory volume in 1 s; FVC, forced vital capacity in 1 s; DLCO, diffusion capacity for carbon monoxide; LAA% = low attenuation areas, lung voxels with Hounsfield Unit (HU) values less than −950; Perc15, HU value at the 15th percentile of the HU value histogram of lung voxels

Quartile analysis revealed a significant association between decreased VE and lower FEV1/FVC (*P*-trend = 0.001), DLCO %predicted (*P*-trend < 0.0001), and Perc15 (*P*-trend = 0.025), while RV/TLC (*P*-trend < 0.0001) and %LAA (*P*-trend = 0.007) were increased (Fig. 2).

In a logistic regression analysis following adjustment for covariates (age, gender, sun exposure, current smoking, and pack year smoking history), the lowest quartile of VE was associated with an increased likelihood of airflow obstruction, adjusted Odds Ratio (OR) 3.66, 95% C.I. 1.49 to 8.99, *P* = 0.005, and visually-assessed pulmonary emphysema, adjusted OR 2.89, 95% C.I. 1.12 to 7.47, *P* = 0.025 (Table 4).

**Table 4:** The lowest quartile of the skin viscoelastic modulus is independently associated with pulmonary emphysema

	VE	Odds Ratio	95% C.I.	*P*-value	*P*-trend
COPD	Median	2.40	1.29 to 4.48	*0.006*	
	Q4 (Ref)	–			
	Q3	1.32	0.58 to 2.97	0.51	*0.004*
	Q2	2.22	0.96 to 5.12	0.063	
	Q1	3.66	1.4*9* to 8.99	*0.005*	
Emphysema	Median	3.27	1.73 to 6.21	*<0.0001*	
	Q4 (Ref)	–			
	Q3	0.69	0.31 to 1.52	0.41	*0.002*
	Q2	2.17	0.95 to 4.95	0.07	
	Q1	2.89	1.12 to 7.47	*0.025*	

Notes: An adjusted logistic regression analysis was performed to test likelihood of emphysema by quartiles of skin viscoelastic modulus (VE) following adjustment for age, gender, sun exposure, current smoking and pack year smoking history. C.I., confidence intervals. The presence of chronic obstructive pulmonary disease (COPD) was defined using FEV1/FVC less than 70%. and subjects were categorized as having emphysema based on a visual emphysema score of greater than zero

### Skin aging is accelerated in individuals with pulmonary emphysema

**Fig. 3:**
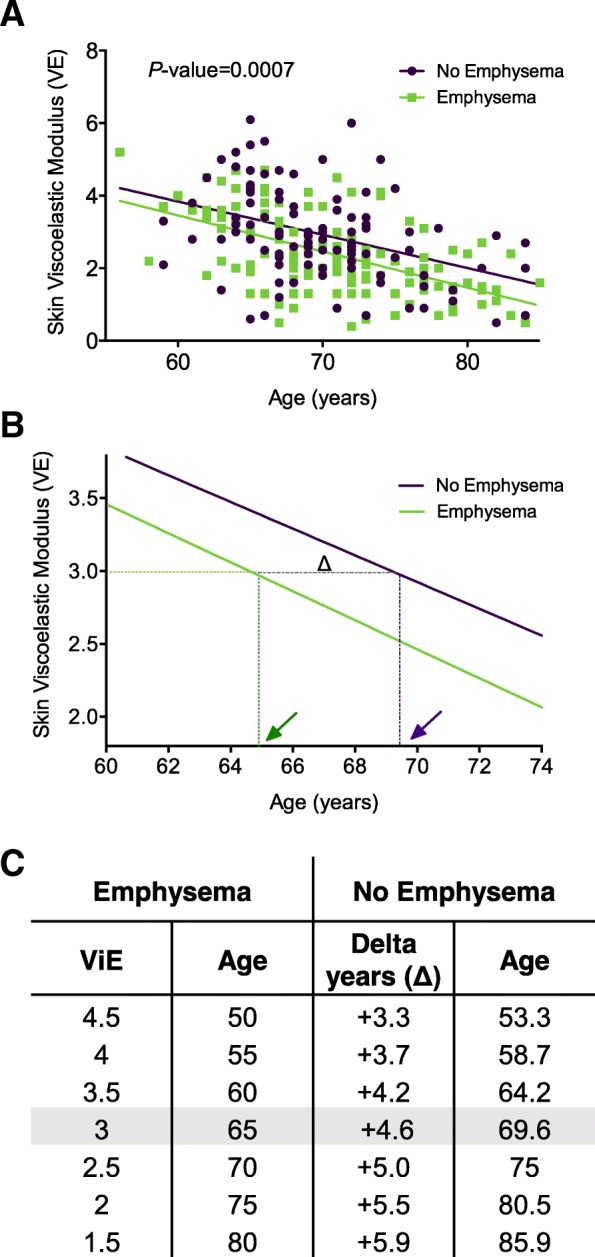
Biological skin aging is accelerated in individuals with pulmonary emphysema. **a** Elasticity-determined skin age was visualized using linear regression modelling of VE against age following stratification for the presence or absence of visually-assessed pulmonary emphysema. **b** Enlarged view of regression model that depicts the linear intercept of VE (value 3.5) with ‘Emphysema’ (green arrow) and ‘No Emphysema’ (purple arrow), the distance between arrows highlights the difference in years between the two groups (Δ). **c** Subjects with visually-assessed pulmonary emphysema had lower skin elasticity at a given chronological age compared to current or former smokers without emphysema, consistent with an increased biological age of skin in the emphysema group. Legend: VE; skin elasticity; Δ, delta/difference

Loss of skin elasticity occurs as a natural consequence of aging and features of increased skin aging have been shown in patients with COPD [13, 28]. After stratification for the presence of pulmonary emphysema, we demonstrated significant differences in skin elasticity at a given chronological age using linear regression modelling, *P* = 0.0007 (Fig. 3a). Elasticity-determined skin age was predicted from the regression model using the linear intercepts of VE with chronological age from subjects with emphysema (Age = (VE-9.433)/− 0.09957) and without emphysema (Age = (VE-9.344)/− 0.09171) (Fig. 3b). Individuals susceptible to emphysema had lower skin elasticity at a given chronological age compared to current or former smokers without emphysema, consistent with an increased biological age of skin in the emphysema group (median difference 4.6 ± 1.3 years, *P* = 0.0007) (Fig. 3c). This finding remained significant after correction for multiple covariates (age, gender, sun exposure, current smoking, and pack year smoking history), subjects with emphysema had a mean reduction in VE of 0.46 (95% C.I. 0.26 to 0.66, *P* = 0.02), which equates to an approximate five-year increase in skin age. There was no interaction between age and emphysema in the adjusted analysis.

### Plasma biomarkers of inflammation and protease activity are associated with skin elasticity

Lower skin elasticity values correlated with increasing plasma biomarkers of systemic inflammation, including the soluble TNFα receptors, TNFR1 and TNFR2, and the acute phase proteins, C-reactive protein (CRP), pentraxin-3 (PTX3), and serum amyloid A (SAA) (Table 5). No correlation was detected in plasma levels of the pro-inflammatory cytokines TNFα and IL-6 with either FWS or VE. Tissue inhibitors of metalloproteases, important regulators of the extracellular matrix, TIMP1, TIMP2, TIMP4, and matrix metalloproteinase 1 (MMP1) were higher in subjects with decreased skin elasticity. In a multiple regression analysis, biomarkers of inflammation, including TNFR2, CRP, and SAA, in addition to TIMP2 and TIMP4 remained significant after correction for current smoking status, pack year smoking history and degree of airflow obstruction (FEV1% predicted).

**Table 5:** The skin viscoelastic modulus is associated with blood biomarkers of inflammation and tissue metalloprotease activity

	Bivariate		Multiple Regression^#^	
	β	*P*-value	β	*P*-value
TNFα				
VE	0.03	0.66	0.18	0.79
FWS	0.012	0.09	0.002	0.98
TNF R1				
VE	*−0.14*	*0.045*	−0.11	0.08
FWS	*0.15*	*0.027*	*0.16*	*0.004*
TNF R2				
VE	*−0.17*	*0.01*	*−0.14*	*0.02*
FWS	*0.136*	*0.049*	*0.15*	*0.02*
CRP				
VE	*−0.15*	*0.03*	*−0.13*	*0.01*
FWS	0.04	0.56	0.04	0.49
PTX3				
VE	*0.21*	*0.002*	0.16	0.051
FWS	0.03	0.63	0.03	0.76
SAA				
VE	−0.16	*0.017*	*−0.15*	*<0.0001*
FWS	0.08	0.25	0.08	0.20
MMP1				
VE	*− 0.16*	*0.02*	*−0.13*	*0.03*
FWS	0.04	0.59	0.05	0.43
MMP7				
VE	−0.09	0.19	−0.08	0.27
FWS	0.11	0.11	0.11	0.08
TIMP1				
VE	*−0.14*	*0.04*	−0.12	0.08
FWS	0.04	0.59	0.04	0.54
TIMP2				
VE	*−0.20*	0.003*	*−0.19*	*0.01*
FWS	0.09	0.18	0.11	0.16
TIMP4				
VE	*−0.22*	0.0015*	*−0.20*	*<0.0001*
FWS	0.09	0.20	0.09	0.21

Notes: Bivariate and multiple regression analyses were performed to evaluate the relationship between plasma biomarkers of inflammation and tissue metalloprotease activity with skin viscoelasticity (VE) and facial wrinkling (FWS)

TNFα, tumor necrosis factor α; TNFR, tumor necrosis factor receptor; CRP, C reactive protein; PTX3, pentraxin 3; SAA, serum amyloid A; MMP, matrix metalloproteinase; TIMP, tissue inhibitor of metalloproteinase

*Significant (*P*<0.05) after correction for multiple comparison testing (Holm-Šídák method). ^#^Adjusted for current smoking, pack year smoking history, and the degree of airflow obstruction

## Discussion

In this study we report that skin elasticity is strongly and independently associated with measures of airflow obstruction and radiographic pulmonary emphysema in a tobacco exposed population. Facial wrinkling did correlate strongly with skin elasticity, though unexpectedly it did not reach a statistically significant association with measures of airflow obstruction or extent of pulmonary emphysema. However, we found a previously unreported direct relationship between facial wrinkling and diffusion impairment, the physiologic correlate to emphysema. Furthermore, we observed that systemic biomarkers of inflammation and metalloprotease activity were inversely associated with skin elasticity, which remained significant after correction for smoking history and degree of airflow obstruction.

The findings of this study validate the concept of extracellular matrix susceptibility to tobacco smoke in the lung and the skin and corroborates the previous findings of increased skin elastosis in biopsy specimens from subjects with COPD compared to matched smokers [19]. Skin elasticity is an accessible and objective determinant of the biomechanical properties of the skin extracellular matrix that has been previously validated in several studies, though hitherto its utility in COPD was unknown [20, 29–32]. As an indirect measure of elastin degradation, skin elasticity reveals a stronger and more convincing association with emphysema than wrinkling and thus represents a viable alternative biomarker. Longitudinal follow up with interval VE measurement may help us determine the impact of continued smoking on skin elasticity and define the predictive value of VE on pulmonary disease progression.

An interesting finding, after stratification for the presence of emphysema, was evidence of more advanced biological aging in the skin of individuals with emphysema. This finding is supported by the well-studied pathological effects of tobacco smoke on non-sun exposed skin, that resemble accelerated aging, whereby dermal elastic fiber size and quantity are increased in smokers compared to age-matched controls without affecting dermal thickness [15, 33]. Notably, in this cross-sectional analysis, decreased skin elasticity was independently associated with emphysema susceptibility at any given chronological age, pointing to the persistent impact of noxious exposure at an earlier phase of the disease course.

Loss of tissue elastic recoil is a key physiological hallmark of pulmonary emphysema that ultimately leads to increased lung compliance, hyperinflation and functional impairment with advancement of disease [34]. Protease-antiprotease imbalance is a key underlying pathological mechanism in COPD and it is known that proteolytically cleaved elastin fibers are pro-inflammatory in the lung [35]. The role of degraded skin elastin contributing to a systemic pro-inflammatory state linked to progression of lung disease is unknown, though studies evaluating the utility of a byproduct of elastin cleavage, desmosine, as a biomarker of pulmonary disease have been inconclusive to date [36].

The proposed mechanism attributed to pathological changes observed in the skin of smokers relates the accumulation of elastin fragments, generated by increased protease activity surrounding the dermal vasculature or driven by toxic effects of tobacco smoke in this region, which may further stimulate elastin and ultrastructural microfibril deposition by dermal fibroblasts [37]. Skin elastin degradation is further increased in the sun-exposed skin of smokers which may belie a synergistic interaction between tobacco smoke exposure and photoaging [19]. It has also been shown that newly synthesized elastin fibers may be rendered defective via inhibition of lysyl oxidase-mediated crosslinking of tropoelastin monomers in the presence of tobacco smoke [38]. Moreover, tobacco smoke exposure is known to exert its toxic effects through a multitude of mechanisms including oxidative stress, free radical damage, pro-inflammatory cytokine release, and the activation of cellular inflammatory response pathways [39]. Persistent systemic inflammation, driven by a heterogeneous array of pathological cellular processes, is an important finding in COPD that is associated with poorer clinical outcomes [40, 41]. Remodeling of the ECM by various MMPs is modulated by pro-inflammatory cytokines such as TNFα, IL-1α, and TGFβ, which are implicated in the pathogenesis of emphysema [42–44]. Though we did not find elevated plasma levels of TNFα, possibly as a result of its short plasma half-life [45], there was a significant association between lower skin elasticity and the more stable soluble TNFα receptors, TNFR1 and TNFR2, which are better indicators for overall activation of the TNFα system [46]. Increased levels of the acute phase reactants CRP and SAA were also associated with decreased skin elasticity that remained significant after correction for smoking history and degree of airflow obstruction.

Mutations in the promoter variants for MMP-1 and MMP-3 represent genetic susceptibility risk factors for an association between facial wrinkling and airflow obstruction [47]. Indicative of shared systems biology, it is notable that cutis laxa, a rare genetic condition caused by mutations in key structural components of elastic fibers (i.e. elastin, fibulin-4, and fibulin-5), manifests with excess inelastic skin and pulmonary emphysema [22]. We found that elevated plasma levels of MMP-1, and cognate tissue metalloproteinase inhibitors, TIMP-1, TIMP3, and TIMP4, were significantly associated with decreased skin VE. Dysregulation of tissue proteinase activity in our cohort is possibly a result of heightened systemic inflammation as shown in our data, or conceivably due to ECM damage with consequent activation of matrikine signaling pathways [48, 49]. There is biological plausibility for the association of MMP/TIMP balance with skin elasticity in our study as it has an important role in the regulation of elastin and collagen degradation of the ECM in the lung [50] and the skin [51]. Hence, loss of skin elasticity may be an important systemic manifestation of inflammatory and extracellular protease pathway activation with implications for matrix degradation in the lung. It is notable that a recent study employing skin autofluorescence measurements revealed an independent association between the accumulation of advanced glycosylation end products (AGEs) in the skin and parameters of lung function in subjects with COPD [52]. In keeping with our findings, the authors reported that AGEs were increased only in smokers susceptible to developing COPD and correlated strongly with the severity of airflow obstruction. Collectively, there is mounting evidence that affirms the biological plausibility underlying the association of pulmonary disease phenotypes with alterations in skin biology in tobacco-exposed individuals.

Given the racial demographics of the University of Pittsburgh SCCOR cohort our study was restricted to Caucasians only, which limits the applicability of our findings to people of a different race but avoids a potential confounding impact on the analysis [53]. Our study cohort differs from Patel et al., who described an association between facial wrinkling and airflow obstruction [13], with respect to an older age of our study population (median 70.4 vs 56.0 years) and thus a higher prevalence and severity of facial wrinkling which may have contributed to reduced variance and a lower power to detect an association with measures of pulmonary function. As we did not directly evaluate skin elastin using invasive or non-invasive techniques, our findings may not relate solely to smoke-related elastolysis but reflect other processes such as accelerated aging due to senescent pathway activation [54].

Finally, accelerated facial aging, altered skin texture, and skin wrinkling may influence a smoker’s decision to quit and are important deterrent factors for the uptake of tobacco products [55]. Our characterization of the linked pathology between degradation of the ECM in the skin and lung and advanced skin aging in those with emphysema, as a consequence of tobacco smoke exposure, may be of importance in public health strategies to enhance tobacco control and aid smoking cessation efforts in the general population.

## Conclusion

This is the first study to associate the biomechanical properties of skin with the severity of airflow obstruction and pulmonary emphysema. Skin elasticity is strongly and independently associated with airflow obstruction, diffusion impairment, gas trapping, and pulmonary emphysema. Moreover, skin aging appears substantially increased in emphysema-susceptible individuals and loss of skin elasticity is associated with elevated blood biomarkers of inflammation and metalloproteinase activity. The findings of our study support the paradigm of complex systemic biological factors in the pathogenesis of COPD and emphysema in those susceptible to the effects of tobacco smoke. Further research into the mechanistic commonality that underlies destruction and remodeling of the ECM, with resultant loss of pulmonary and cutaneous elasticity, may help elucidate common pathological processes and lead to future developments in the field of emphysema and COPD research.

## Additional file

Additional file 1: **Figure S1.** Measurement of Skin Elasticity. A) Skin Elastance was measured on the volar forearm using the Dermalab® skin elasticity unit. Increasing increments of negative pressure were applied to a section, 10 mm in diameter, measuring skin distension in the elevation phase and, upon release of the vacuum, rate of return during the retraction phase. (PPTX 618 kb)

## Data Availability

The datasets used and/or analyzed during the current study are available from the corresponding author on reasonable request.

## Abbreviations

AGEs: advanced glycosylation end products
BMI: body mass index
COPD: chronic obstructive pulmonary disease
CRP: C reactive protein
CT: computerized tomography
DLCO: diffusion capacity for carbon monoxide
E: Young’s elastic modulus
ECM: extracellular matrix
FEV1: forced expiratory volume in 1 s
FVC: forced vital capacity in 1 s
FWS: facial wrinkling score
GOLD: Global initiative for obstructive lung disease
HU: Hounsfield unit
IL-1α: interleukin-1 alpha
IQR: interquartile range
LAA%: low attenuation areas, lung voxels with Hounsfield Unit (HU) values less than − 950
MMP: matrix metalloproteinase
Perc15: HU value at the 15th percentile of the HU value histogram of lung voxels
PTX3: pentraxin 3
r: radius
RT: retraction time
RV: residual volume
SAA: serum amyloid A
SCCOR: specialized center of clinically orientated research
SD: standard deviation
TGFβ: transforming growth factor beta
TIMP: tissue inhibitor of metalloproteinase
TLC: total lung capacity
TNFR: tumor necrosis factor receptor
TNFα: tumor necrosis factor alpha
VE: skin elasticity
VE: skin viscoelastic modulus
Δ: delta/difference
