# Supplementary figures and images for: Loss of skin elasticity is associated with pulmonary emphysema, biomarkers of inflammation, and matrix metalloproteinase activity in smokers
Source: Respir Res. 2019 Jun 24;20:128. doi: 10.1186/s12931-019-1098-7 (PMC6591816; doi:10.1186/s12931-019-1098-7)

## Slide 1
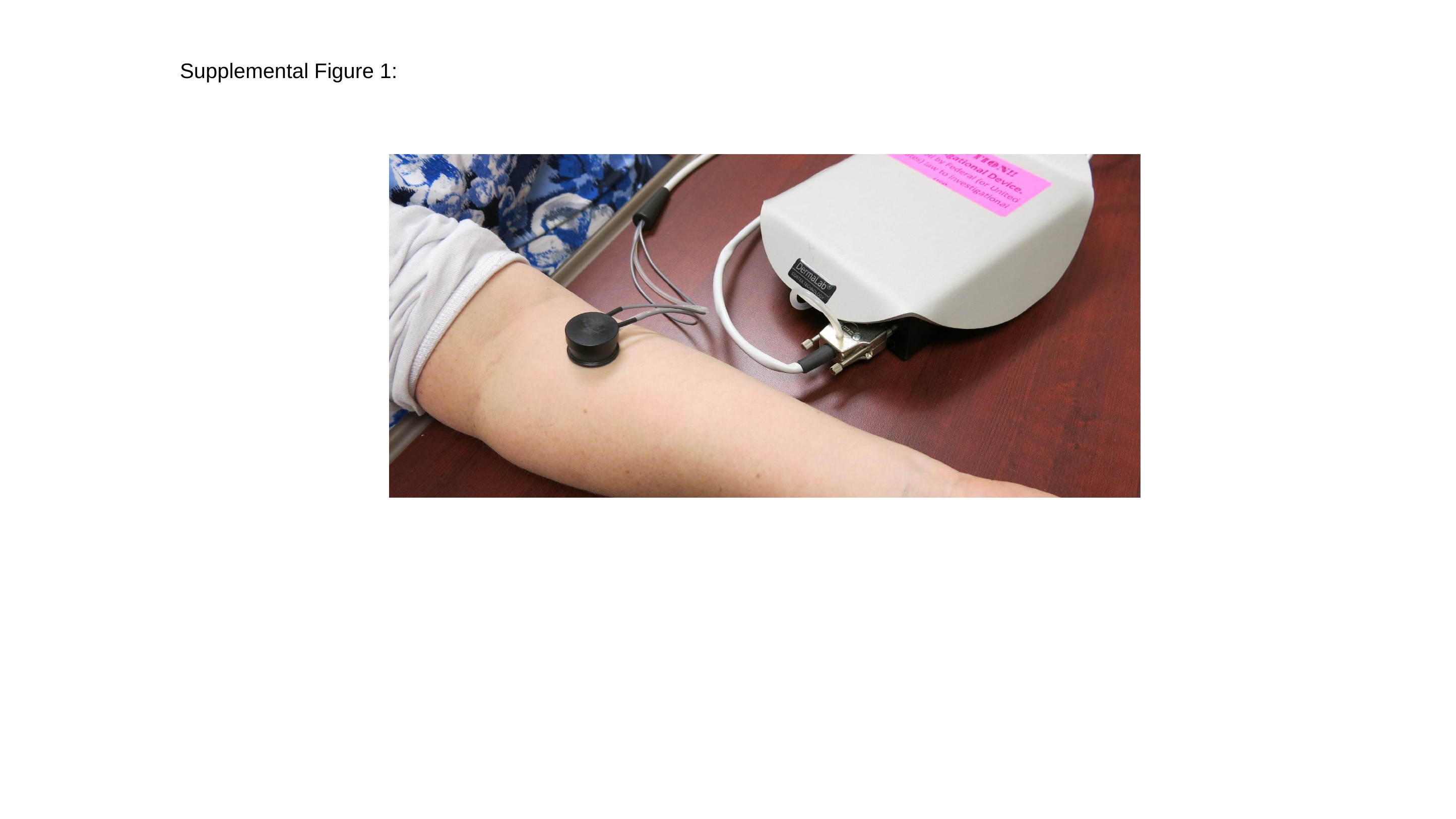

Supplemental Figure 1:

Supplement: Supplementary file 1 — Figure S1. Measurement of Skin Elasticity. A) Skin Elastance was measured on the volar forearm using the Dermalab® skin elasticity unit. Increasing increments of negative pressure were applied to a section, 10 mm in diameter, measuring skin distension in the elevation phase and, upon release of the vacuum, rate of return during the retraction phase. (PPTX 618 kb) [file 12931_2019_1098_MOESM1_ESM.pptx]
